# Editorial: Advances in molecular and pharmacological mechanisms of novel targeted therapies for melanoma

**DOI:** 10.3389/fonc.2024.1403778

**Published:** 2024-04-04

**Authors:** Marcel Henrique Marcondes Sari, Luana Mota Ferreira

**Affiliations:** ^1^ Departamento de Análises Clínicas, Universidade Federal do Paraná, Curitiba, Paraná, Brazil; ^2^ Programa de Pós-Graduação em Ciências Farmacêuticas, Universidade Federal do Paraná, Curitiba, Paraná, Brazil

**Keywords:** skin cancer, melanoma, immunology, nanotechnology, cancer biomarkers, drug repositioning, immunotherapy

Over the years, extensive research efforts have been made to identify specific biomarkers that can predict the growth and behavior of melanoma cells, with the ultimate goal of developing targeted therapies that can effectively treat this disease. To address this challenge, researchers are actively working to develop new and improved targeted therapies. Collectively, eleven studies were published by authors from China (7/11), United States (2/11), Germany (1/11), and Italy (1/11), approaching novel targets for melanoma diagnosis and prognosis, and promising therapeutic interventions to counteract the disease.

Ferroptosis could be an effective way to prevent malignant melanoma (MM) development. In the review of Ta et al., a comprehensive overview of the fundamental mechanisms underlying the ferroptosis development in MM cells and its potential as a therapeutic target were proposed. Inducing ferroptosis is generally considered an effective approach to induce cell death in therapy-resistant MM cells. Additionally, nano-based medicines have shown promise in inducing the ferroptosis pathway in MM. Aiming to comprehend the mechanisms underlying cinobufagin action in melanoma, Yang et al. combined network pharmacology with other sequencing data to identify key targets. Results showed that cinobufagin may exert its effects by halting the cell cycle through three protein tyrosine/serine kinases (EGFR, ERBB2, and CDK2) inhibition. Liu et al. developed a new Mitogen-activated protein kinase (MEK) inhibitor, the tunlametinib, which exhibits high *in vitro* selectivity and shows significant potency against RAS/RAF mutant cells. *In vivo* studies demonstrate that tunlametinib has a favorable pharmacokinetic profile and leads to significant tumor suppression. When combined with BRAF/KRASG12C/SHP2 inhibitors or docetaxel, tunlametinib exhibits a synergistic response and substantial tumor inhibition.

Wan et al. employ bibliometrics to analyze research on biomarkers in melanoma, offering insights into the field’s historical development, current status, and future research directions. Findings reveal a steady increase in both the number of publications and citation frequency in this area, with a notable surge in citation frequency observed post-2018. Biomarkers associated with melanoma diagnosis, treatment, and prognosis are identified as key topics and cutting-edge areas of interest within the field. Another study conducted by Tao et al. explored public databases to prospect potential therapeutic targets for BRAF-mutated melanoma. A total of 24 overlapping genes were identified by analyzing differentially expressed genes common to melanoma and non-transformed tissue, BRAF-mutated and BRAF wild-type melanoma. Among them, (cytokine-like 1) CYTL1 was highly expressed in melanoma, especially in BRAFmutated melanoma, and the high expression of CYTL1 was associated with epithelial-mesenchymal transition, cell cycle, and cellular response to ultraviolet radiation. In melanoma patients, clinical studies showed a positive correlation between increased CYTL1 expression and shorter overall survival and disease-free survival. Lastly, the authors confirmed that the knockdown of CYTL1 significantly inhibited the migration and invasive ability of melanoma cells by conducting *in vitro* assessments.

Immune checkpoint inhibitors (ICI) are increasingly utilized in the treatment of melanoma. However, a recognized complication associated with these inhibitors is colitis, often managed with medical treatment. In this sense, Childers et al. presented a case involving a patient with stage IV melanoma undergoing ICI therapy. While ICIs-induced colitis can occur in patients with various types of cancer, the majority of existing evidence is predominantly associated with melanoma. This is because ICIs are commonly employed as the primary treatment for advanced-stage melanoma. While mild side effects can be monitored and treated symptomatically, severe immune-related adverse effects may progress rapidly, leading to complications requiring surgical intervention. The authors concluded that further research is warranted to comprehend the incidence of colitis progression in the context of single and multiple ICI combinations for malignancy. Similarly, Reiter et al. discussed a case report of a patient with metastatic Uveal melanoma (UM) who initially experienced extensive progression while receiving tebentafusp treatment but later exhibited a remarkable response to combined ICI therapy. Recently, a bispecific gp100 peptide-HLA-directed CD3 T cell engager known as tebentafusp has gained approval for the treatment of unresectable UM. Despite its complex treatment regimen involving weekly administrations and close monitoring, the response rate remains limited. Moreover, there is limited data available on the use of combined ICIs in UM following prior progression on tebentafusp. In cases where progressive findings are observed during the initial follow-up, continuation with tebentafusp should be considered. However, in the event of further progression of metastases, treatment with combined ICI may be a viable option. Lastly, Deng et al. investigated the correlation between Integrin Subunit Alpha L (ITGAL) expression and immune infiltration, clinical prognosis, and specific T cell types in melanoma tissue. The results underscore the pivotal role of ITGAL in melanoma and its potential mechanism in regulating tumor-infiltrating immune cells, rendering it a promising diagnostic biomarker and therapeutic target for advanced melanoma. The findings reveal that heightened ITGAL expression correlates not only with PD1 and CTLA4 but also with other potential melanoma checkpoints, suggesting that ICIs have become melanoma treatment cornerstone, with PD1/PDL1 checkpoint blockade therapy being a standard approach.

Oligometastatic progression is a challenge in tumor immune evasion that occurs when tumors spread to a limited number of distant sites. This type of progression can be difficult to diagnose and manage using ICIs. A potential solution is to use circulating tumor DNA (ctDNA) alongside surveillance imaging for diagnosis and disease monitoring. Khaddour et al. discussed two cases of patients, one with metastatic melanoma and the other with metastatic Merkel cell carcinoma, who underwent ICI therapy and subsequently developed localized resistance due to oligometastatic progression. In response, stereotactic body radiation therapy (SBRT) was employed as a salvage approach to address the oligometastatic progression. Furthermore, the study elucidates the temporal and dynamic relationship of ctDNA before, during, and after SBRT, strongly suggesting the diagnosis without the need for obtaining a histological specimen.

The relationship between serum 25-Hydroxyvitamin D (25HVD) levels and the incidence of melanoma using Mendelian randomization (MR) was investigated by Cai et al. The MR analysis revealed a significant positive causal relationship between serum 25HVD levels and cutaneous melanoma incidence, suggesting that the risk of developing melanoma increases with each unit increase in serum 25HVD concentration. Remarkably, a potentially causal positive association between serum 25HVD levels and melanoma risk, challenging traditional beliefs about vitamin D’s role in melanoma. Metastatic dissemination stands as a primary contributor to mortality in melanoma patients. In this sense, KiSS1 holds significant given its application as epigenetic agent, and it can exhibit a pro-apoptotic effect when combined with cisplatin. Guzzetti et al. demonstrated a significant augmentation of vemurafenib’s pro-apoptotic activity by kisspeptin 54, a peptide derived from KiSS1 cleavage, even in cellular models resistant to the drug. The efficacy of this combination therapy appears to hinge on the intrinsic susceptibility of each cell line to drug-induced apoptosis.

## Author contributions

MS: Writing – original draft, Writing – review & editing. LF: Writing – original draft, Writing – review & editing.

